# Mechanisms of *Yersinia* YopO kinase substrate specificity

**DOI:** 10.1038/srep39998

**Published:** 2017-01-04

**Authors:** Wei Lin Lee, Pavithra Singaravelu, Sheena Wee, Bo Xue, Khay Chun Ang, Jayantha Gunaratne, Jonathan M. Grimes, Kunchithapadam Swaminathan, Robert C. Robinson

**Affiliations:** 1 Institute of Molecular and Cell Biology, A*STAR (Agency for Science, Technology and Research), Singapore; 2 Department of Biological Sciences, National University of Singapore, Singapore; 3 Department of Anatomy, National University of Singapore, Singapore; 4 Division of Structural Biology, Wellcome Trust Centre for Human Genetics, University of Oxford, UK; 5 Diamond Light Source Ltd., UK; 6 Department of Biochemistry, National University of Singapore, Singapore; 7 NTU Institute of Structural Biology, Nanyang Technological University, 59 Nanyang Drive, 636921, Singapore; 8 School of Biological Sciences, Nanyang Technological University, 60 Nanyang Drive, 637551, Singapore; 9 Lee Kong Chan School of Medicine, 50 Nanyang Avenue, 639798, Singapore

## Abstract

*Yersinia* bacteria cause a range of human diseases, including yersiniosis, Far East scarlet-like fever and the plague. *Yersiniae* modulate and evade host immune defences through injection of *Yersinia* outer proteins (Yops) into phagocytic cells. One of the Yops, YopO (also known as YpkA) obstructs phagocytosis through disrupting actin filament regulation processes - inhibiting polymerization-promoting signaling through sequestration of Rac/Rho family GTPases and by using monomeric actin as bait to recruit and phosphorylate host actin-regulating proteins. Here we set out to identify mechanisms of specificity in protein phosphorylation by YopO that would clarify its effects on cytoskeleton disruption. We report the MgADP structure of *Yersinia enterocolitica* YopO in complex with actin, which reveals its active site architecture. Using a proteome-wide kinase-interacting substrate screening (KISS) method, we identified that YopO phosphorylates a wide range of actin-modulating proteins and located their phosphorylation sites by mass spectrometry. Using artificial substrates we clarified YopO’s substrate length requirements and its phosphorylation consensus sequence. These findings provide fresh insight into the mechanism of the YopO kinase and demonstrate that YopO executes a specific strategy targeting actin-modulating proteins, across multiple functionalities, to compete for control of their native phospho-signaling, thus hampering the cytoskeletal processes required for macrophage phagocytosis.

Pathogenic *Yersinia* species give rise to a number of human diseases. *Y. enterocolitica* causes gastrointestinal syndromes; *Y. pseudotuberculosis*, Far East scarlet-like fever; and *Y. pestis* is notorious for pandemics, the causative agent of the plague^1^. During infection, pathogenic *Yersinia* species use a type III secretion system to inject a cocktail of six effectors into the host cell. These proteins, known as *Yersinia* outer proteins (Yops), collectively inhibit the phagocytosis machinery and modulate host signaling to reorient the innate immune response of the host^2^. Four of these proteins target the host actin cytoskeleton – YopH is a tyrosine phosphatase that disrupts focal adhesion complexes^3^ while YopE, YopT and YopO target Rho GTPases, albeit in different ways. YopT is a cysteine protease which cleaves and releases Rho GTPases from their sites of action at the membrane^4^, YopE is a GTPase-activating protein, turning off the signaling capacity of Rho GTPases^5^, and YopO, also known as YpkA, contains a guanidine nucleotide dissociation inhibitor (GDI)-like domain^6^.

Besides a GDI-like domain, YopO also harbors an N-terminal membrane localization domain and a serine/threonine kinase domain^7^. YopO, when overexpressed in cells in culture, gives rise to phenotypes typified by the disruption of stress fibres and cell edge retraction^8^,^9^,^10^. YopO inhibits Fcγ-mediated phagocytosis^11^ and YadA-dependent bacterial uptake in an actin-dependent manner^12^. YopO’s kinase activity plays an important role in virulence as seen in a mouse model in which *Yersinia pseudotuberculosis* carrying kinase-dead YopO is attenuated in infectiousness with respect to wild-type bacteria^13^. Recombinant and *Yersinia* secreted YopO has no kinase activity^14^,^15^ until it binds to monomeric actin present within the eukaryotic host cell^9^. The serine/threonine kinase domain and the GDI-like domain sandwich actin, triggering YopO autophosphorylation^12^ and the allosteric stabilization of the active state of the kinase active site^16^. In this conformation, the catalytic cleft lies at the interface of the kinase domain and the GDI domain, and positions proximal to subdomain 4 of the bound actin^16^. YopO interacts with actin-modulating proteins through actin, forming ternary complexes. Some of these proteins are found to be phosphorylated by YopO, including Ena/VASP family proteins, DIAPH1, WAS and gelsolin^16^.

Here we endeavor to identify mechanisms conferring specificity in protein phosphorylation by YopO that clarify the effects of the YopO kinase on cytoskeleton disruption. We report the X-ray structure of MgADP YopO:actin complex, which reveals that YopO is most structurally similar to mammalian p21-activated kinases (PAKs). We identify, using a proteome-wide kinase-interacting substrate screening (KISS) method, that YopO phosphorylates a range of actin-modulating proteins, and report their phosphorylation sites. We analyze determinants for phosphorylation by YopO using artificial substrates to determine the length required to span from the kinase catalytic cleft to the actin-binding site and also determine that YopO may phosphorylate sequences recognized by PAKs and to lower extents protein kinase C (PKC) and protein kinase A (PKA). Thus, our findings provide understanding into the phosphorylation mechanisms of YopO and support the hypothesis that YopO may hijack native host phosphorylation pathways to divert control of actin-cytoskeletal dynamics during infection.

## Results

### Structure of MgADP YopO:actin complex

**Figure 1:**
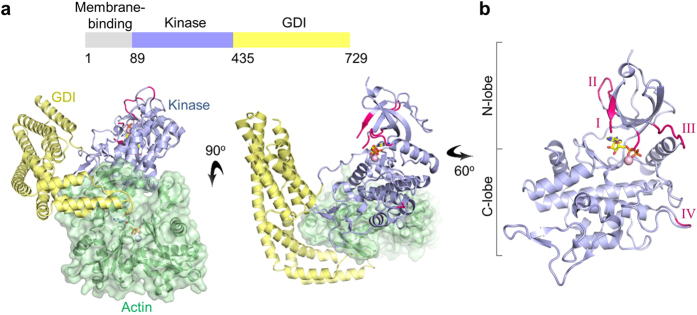
Crystal structure of YopO-actin:MgADP. **(a)** Crystal structure of the kinase (light blue) and GDI (pale yellow) domains in complex with actin (pale green). MgADP lies in the active site of the YopO kinase domain. **(b)** Structure of the kinase domain of YopO. Regions that are ordered in this ADP-bound structure, but were disordered in the apo structure (PDB: 4CI6), are colored pink and labeled I–IV with reference to Supplementary Fig. 1.

We first sought to understand the kinase active site architecture of YopO, by determining the X-ray structure of YopO:actin in complex with Mg^2+^ and ATP (Fig. 1 and Supplementary Table 1). Inspection of the resultant electron density map revealed that the ATP had undergone hydrolysis to form ADP. MgADP binding to YopO stabilizes the active site of the kinase domain via interactions with the P-loop and its surrounding residues. Many of the loops that were disordered in the YopO:actin apo structure^16^ become ordered on binding to MgADP (Supplementary Fig. 1). In the YopO-actin:MgADP structure, residues 135–138, 143–144, 150–155 and 162–167 in the N-lobe gain order, while residues 139–142 of the ATP-binding loop (P-loop) remain disordered. In the C-lobe, residues 313–314, adjacent to the APE (Ala-Pro-Glu) motif in the activation segment, also become ordered in the presence of MgADP. Residues 510–529 of the GDI domain remain disordered in both the nucleotide-bound and nucleotide-free structures.

Using the structure comparison tool Dali^17^, we compared the kinase domain of YopO (residues 109–434) with the solved structures of all kinases in Protein Data Bank. The kinase domain of YopO is structurally more closely related to known human serine/threonine kinase structures than to bacterial kinase structures (Supplementary Fig. 2). Despite the low sequence identities (<18%), mammalian p21-activated kinase PAK1 and mammalian STE20-like protein kinase 3 (MST3) have RMSDs for equivalent Cα atoms of between 2.6 and 2.8 Å for pairwise comparisons with the YopO kinase domain^18^. We went on to compare structural features of the kinase domain of YopO with those of PAK1 and MST3. Exemplified by the low sequence identity, different hydrophobic residues line the adenine-binding pocket in comparison to PAK1 and MST3, while the overall active site architecture of the kinase domain of YopO is structurally conserved (Supplementary Fig. 2c).

**Figure 2:**
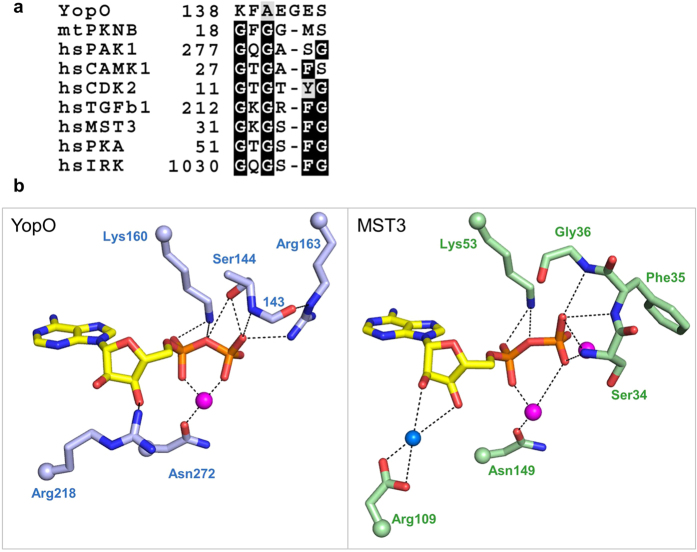
Coordination of ADP by the non-consensus YopO P-loop. **(a)** Sequence alignment of P-loop sequences across kinases from different families. Full alignments can be found in Supplementary Fig. 2. **(b)** Hydrogen-bonding interactions that coordinate the kinase-bound ADP. Magenta spheres represent Mg^2+^ ion in YopO and Mn^2+^ ions in MST3 (PDB: 3A7J) while blue spheres represent water. For clarity, only the side chain is shown for some residues while their attachment to the main chain is represented as a sphere, and the side-chain atoms of residue 143 are not shown.

The P-loop of most kinases consists of a conserved glycine-rich motif GXGXφG (where φ is usually tyrosine or phenylalanine) in which the glycine mainchain amides coordinate the phosphates of ATP. We observed in the YopO structure that the P-loop comprised of a non-consensus motif 138-KFAEGES-144 (Fig. 2a and Supplementary Fig. 3a). We compared the coordination of ADP by YopO with that of MST3-ADP, which contains a canonical motif GKGSFG (Fig. 2b). While only the first and last two residues of YopO’s P-loop were ordered, we observed differences in the coordination of the β-phosphate of ADP. In MST3, the backbone amides of Ser34, Phe35 and Gly36 of the P-loop coordinate the β-phosphate of ADP. However, in YopO, while the backbone amide of Ser144 coordinates the β-phosphate of ADP, the side chain hydroxyl of Ser144 and the side chain guanidinium of Arg163, which is not in the P-loop, also contribute to coordinating the β-phosphate. In MST3, the hydroxyl groups of the ribose are coordinated through water, but in YopO, Arg218 directly coordinates one of the hydroxyl groups. Other features remain similar–the coordination of the metal ion through an Asn and the α-phosphate through a Lys.

Most protein kinases contain the DFG motif at the start of the activation segment. In contrast, YopO contains an unconventional DLG motif, similar to TGFβ kinases (Supplementary Fig. 3a). YopO has an uncharged residue – Asn272 instead of the usual arginine (R), which immediately precedes the catalytic aspartate (D) of the catalytic loop. Non-RD kinases are known to have their activation loops adopt open and extended conformations in the absence of phosphorylation^19^. While no noticeable electron density corresponding to phosphate groups was present in the final electron density maps, mass spectrometry analysis detected phosphorylation at either Thr301 or Ser303 within the activation loop (Supplementary Fig. 3b). At the N-terminus of the kinase domain, besides Ser90 and Ser95^12^, we also observed autophosphorylation at Ser102. Ser102 and/or Thr301/Ser303 may account for observations of residual autophosphorylation detected for the YopO S90A/S95A mutant^12^. The activation loop phosphorylation site is usually involved in stabilizing the active conformation through simultaneous coordination of the activation loop, catalytic loop, αC-helix and the αE/αF linker from the C-lobe^19^. However, neither Thr301 nor Ser303 appears to be involved in such extensive coordination, suggesting that this residual phosphorylation may have little physiological importance.

### Identification of substrates by kinase-interacting substrate screening

**Figure 3:**
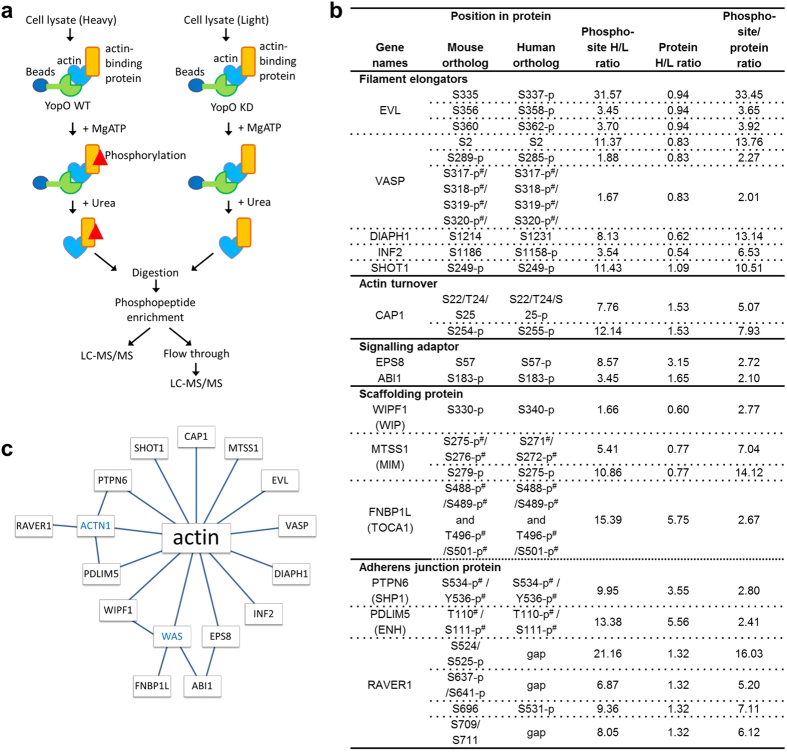
Screening for YopO substrates using the KISS method. **(a)** Scheme of the KISS method. **(b)** Proteins identified as YopO kinase substrates. Phosphorylation sites with phosphosite/peptide ratio above 2 were considered as putative YopO phosphorylation sites. The phosphosites were further subjected to cut-offs of mass spectrometry identification score of 44 and protein ratio count of 4. Alternative names for the proteins were shown in brackets. KISS was performed using mouse monocyte/macrophage cell line Raw264.7. Phosphorylation sites and their corresponding residues in the human ortholog are shown. p denotes reported phosphorylation of the human or mouse ortholog according to Phosphosite^45^. # denotes ambiguous identification in MS due to close proximity of potential phosphorylation sites. **(c)** Model of the network of direct and indirect interactions of YopO substrates to actin. Proteins shown in blue were detected in KISS but were not found to be significantly phosphorylated.

We performed a proteome-wide screen for substrates of YopO’s kinase activity with modifications to the method KISS^20^ to enable relative protein quantitation with the inclusion of stable isotope labeling with amino acids in cell culture (SILAC). KISS allows the identification of kinase substrates and their phosphorylation sites on the basis of kinase–substrate complex formation. YopO WT coated affinity beads were allowed to pull down interactors from the heavy isotope macrophage-like Raw264.7 cell lysate and then incubated with ATP, Mn^2+^ and Mg^2+^ to permit phosphorylation. A parallel experiment was performed for YopO KD (kinase dead mutant, D267A K269A) against light isotope lysate as a control (Fig. 3a) to sidestep the issue of phosphorylation generated by endogenous kinases in the cell lysate. We previously demonstrated that YopO KD does not autophosphorylate or phosphorylate substrates^16^. The samples were digested, and the phosphorylated peptides were concentrated using an IMAC column, followed by analysis by LC-MS/MS. The fold change of each phosphorylation site in the heavy over the light state is indicated by “phosphosite H/L ratio normalized” (Fig. 3b). The flow-through from the IMAC column was retained and analyzed in a separate LC-MS/MS analysis. Peptides belonging to 1412 proteins were identified and 1937 phosphorylation sites were detected. The relative abundances of proteins as calculated by their compositional peptides are indicated by “protein groups H/L ratio normalized”. The distributions of quantified phosphorylation sites, proteins and the phosphorylation site/protein ratio can be found in Supplementary Fig. 4. Most proteins are pulled down in similar amounts between YopO WT and KD, and as such most of their ratios are approximately unity. The peptide/protein ratio is then calculated to derive the fold change of a particular phosphorylation site normalized by the fold change of protein.

The KISS results reveal that YopO phosphorylates a wide range of actin-binding proteins, including: filament elongators EVL and VASP, formins DIAPH1 and INF2; actin-dependent neuronal polarization protein SHOT1; actin turnover protein CAP1; signaling adaptor proteins EPS8 and ABI1; scaffolding proteins WIPF1, MTSS1 and FNBP1L; and adherens junction proteins PTPN6 and PDLIM5. A number of nucleic-acid-binding proteins also appear to be phosphorylated by YopO. These may be due to non-specific interactions with contaminating nucleic acids^21^ (Supplementary Table 2), although one of these proteins, RAVER1, a heterogeneous nuclear ribonucleoprotein, has been reported to play a role in cytoskeletal architecture through interactions with α-actinin at focal adhesions^22^. Some of these proteins like ABI1 and FNBP1L have not been reported to be direct binders of actin and may have been recruited to YopO through proteins that directly bind actin (Fig. 3c). FNBP1L may be recruited to YopO through binding WAS^23^, while ABI1 may be recruited either through EPS8, as a tri-complex with SOS-1^24^, or through WAS. WAS and α-actinin were identified in KISS but no phosphorylated sequences were detected.

**Figure 4:**
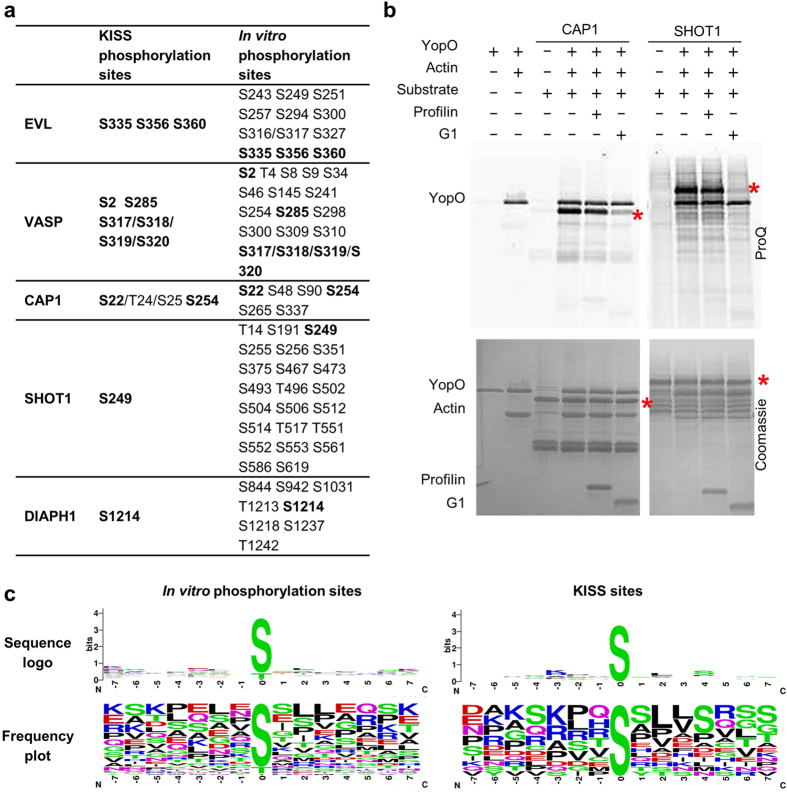
*In vitro* phosphorylation of actin-binding proteins by YopO. (**a**) Mass spectrometry identification of phosphorylation sites obtained by *in vitro* phosphorylation. Human EVL, VASP and SHOT1 and mouse CAP1 and DIAPH1 (residues 583–1255) were used. Residues numbering follows the mouse sequences for clarity. (**b**) *In vitro* phosphorylation assay, monitored by sequential ProQ Diamond phosphoprotein and Coomassie staining. Red asterisks indicate the molecular weights of the substrates. Final protein concentrations used in the assay were YopO (1.1 μM), G-actin (1.1 μM), substrates (5.5 μM), G1 (3.3 μM) and profilin (3.3 μM). **(c)** Preferred YopO phosphorylation motifs, computed from sequences in (Supplementary Fig. 9).

To confirm that the phosphorylation sites identified by KISS were directly phosphorylated by YopO, a subset of these proteins were recombinantly expressed and subject to phosphorylation using a purified system of YopO, actin, substrate and 3-fold excess profilin, followed by LC-MS/MS analysis (Fig. 4a and Supplementary Table 3). Profilin is included to simulate native binding modes since most of the available monomeric actin in the cell exists in a complex with profilin^25^. Many more phosphorylation sites were detected using these purified protein assays than were identified with the KISS methodology. This could arise from promiscuous activity of YopO in a purified system, as well as improved mass spectrometry detection due to the decrease in sample complexity. All phosphorylation sites identified by KISS were recapitulated with this *in vitro* phosphorylation. A tabulation of validated YopO phosphorylation sites shows that YopO strongly favors serine over threonine as the phosphoacceptor and it does not phosphorylate tyrosine residues.

These mass spectrometry data confirm that SHOT1 is phosphorylated by YopO at Ser249, which contains the canonical PAK phosphorylation sequence (K/R-R-X-p[S/T])^26^. Given the structural similarity of YopO’s kinase domain with PAKs, we assayed whether PAKs (in this case PAK4) can phosphorylate other substrates of YopO (Supplementary Fig. 5). Except SHOT1, PAK4 was not observed to significantly phosphorylate any of the other YopO substrates, suggesting that while YopO may have some substrate overlap with PAKs, YopO has a different substrate selection strategy to that of the PAKs.

We have previously reported that the phosphorylation of EVL, VASP and DIAPH1 requires their recruitment by actin, and/or profilin-actin^16^. Here we asked whether the phosphorylation of CAP1 and SHOT1 occurs through the same mechanism. We tested the phosphorylation of CAP1 and SHOT1 *in vitro* in the presence/absence of excess profilin or gelsolin domain 1 (G1). Their phosphorylation were unaffected by the presence of excess profilin but were diminished in the presence of excess G1 (Fig. 4b). Profilin binds between subdomains 1 and 3 on actin (K_d_ = 0.7 μM)^27^ in a way that can accommodate proteins like SHOT1 or CAP1, which contain profilin-binding polyproline motifs, in a quaternary complex with YopO-actin. G1 binds to actin likewise between subdomains 1 and 3 (K_d_ = 5 pM), however in a manner excluding the binding of other actin-binding proteins in the same pocket^28^. These data show that these substrates depend on their engagement by actin, and/or profilin–actin, in order to be presented to the kinase domain for phosphorylation.

### Identification of mode of binding

Since phosphorylation by YopO depends on substrate recruitment by actin, we mapped out the locations of phosphorylation on each protein (Supplementary Fig. 6). We observed that proteins which possess similar domain organization are phosphorylated at similar regions. VASP and EVL share phosphorylation sites between the F-actin binding domain and the tetramerization domain suggesting that these sites may be targeted in a similar manner as they bind to actin (Supplementary Fig. 7). DIAPH1, like INF2, is phosphorylated C-terminal to the DAD domain.

We performed disorder prediction (PONDR) for the YopO substrates and identified that the regions in which the phosphorylation sites are present are predicted to be disordered (Supplementary Fig. 8). This is consistent with structural models constructed between YopO, actin and actin-binding proteins^16^, in that only substrate regions that can access the catalytic cleft flanked between the kinase domain and the GDI domain become phosphorylated. The kinase and the GDI domain are positioned just 33 Å apart^16^, physically restricting access of bulkier substrate regions, suggesting that substrate tertiary structure will affect the propensity for phosphorylation by YopO.

We compared the phosphorylation sites derived from *in vitro* phosphorylation and KISS. For the former, there appeared to be low or no sequence specificity flanking the phosphoacceptor residue. Amongst the KISS phosphorylation sites, basic residues appear more frequently at the −3 position and small hydrophobic residues at positions +2 and +3 and serine at the +4 position (Fig. 4c and Supplementary Fig. 9).

### Specificity determinants of protein phosphorylation by YopO

**Figure 5:**
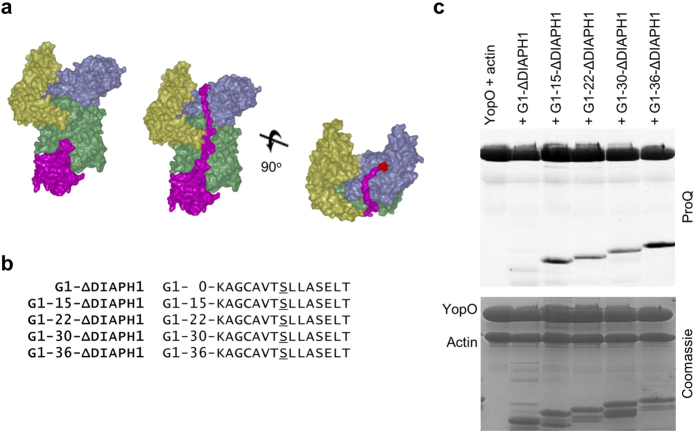
Length requirement from actin-binding site to kinase catalytic cleft. **(a)** Model for phosphorylation of recruited actin-binding proteins by YopO. Model 1 is a superimposition of gelsolin domain 1 up to FKHV (28–152) from the G1-G3:actin complex (PDB:3FFK). Model in middle and right show an extension of 26 residues reaching up into the catalytic cleft of YopO with active site residues in red. **(b)** Artificial substrates of YopO with G1 fused to a phosphorylation sequence from DIAPH1 with the insertion of poly glycine-alanine linkers of different lengths between G1 and the phosphorylation sequence. **(c)** *In vitro* phosphorylation assay of artificial substrates of various lengths. The SDS-PAGE gel was sequentially imaged with ProQ Diamond phosphoprotein and Coomassie staining. Final protein concentrations were YopO (1.8 μM), G-actin (1.8 μM), artificial substrates (5.4 μM).

Together with our previous work, we have shown that amongst the actin-binding proteins tested, YopO does not phosphorylate G1, CapG, twinfilin and profilin^16^. We hypothesize that the propensity for phosphorylation depends on the ability of the substrate to stretch from the actin-binding site to the catalytic cleft of the kinase. As such we designed artificial substrates to determine the length requirement for presentation to the kinase domain (Fig. 5 and Supplementary Table 4). We made an artificial substrate by fusing G1 to a sequence from the C-terminus of DIAPH1 that we determined was phosphorylated by YopO and included seven residues upstream and downstream of the phosphorylated serine. Poly-glycine-alanine linkers of different lengths were inserted between G1 and the phosphorylation sequence and these were then assayed for their ability to be phosphorylated. G1-15-ΔDIAPH1 but not G1-ΔDIAPH1 is phosphorylated, suggesting that, assuming the absence of secondary structure, the minimum length requirement to reach from the end of the FKHV motif to the kinase active site lies between 18–32 residues. Constructs G1-15-ΔDIAPH1 up to G1-36-ΔDIAPH1 were all phosphorylated, suggesting that while there is a minimum length requirement, YopO accepts longer substrates of up to the 53 residues in G1-36-ΔDIAPH1.

**Figure 6:**
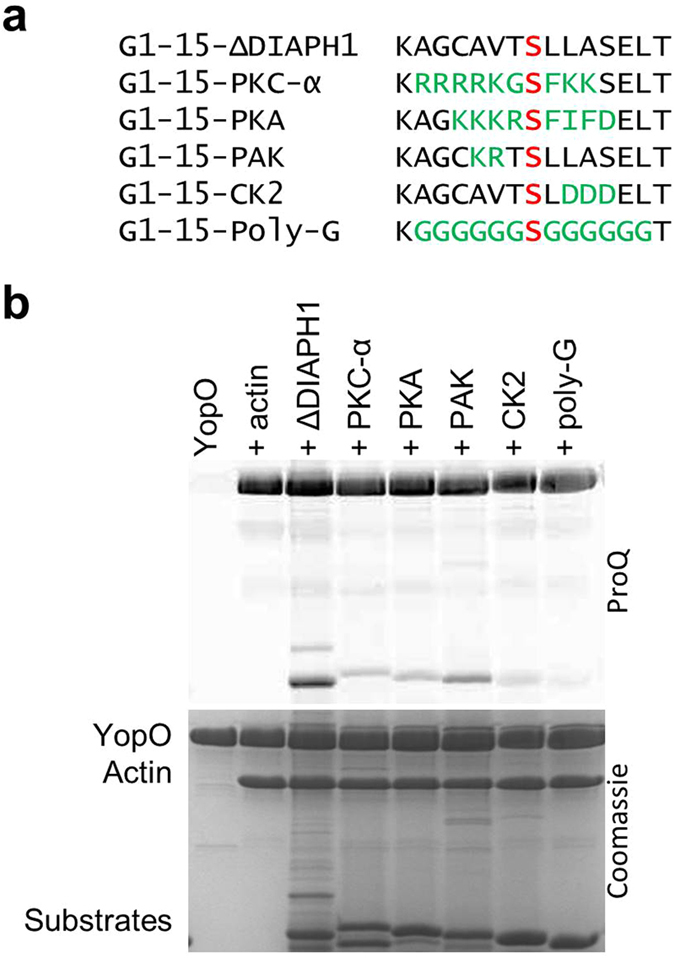
Recognition of phosphorylation motifs of other kinases by YopO. (**a**) Artificial substrates containing phosphorylation motifs belonging to different kinases. (**b**) *In vitro* phosphorylation assay. The SDS-PAGE was sequentially imaged with ProQ Diamond phosphoprotein and Coomassie staining. Final protein concentrations were YopO (1.8 μM), G-actin (1.8 μM), artificial substrates (5.4 μM).

To clarify the consensus recognition sequence of YopO, we asked if YopO could target consensus sequences recognized by different kinase families. The phosphorylation sequences in G1-15-ΔDIAPH1 were substituted with consensus sequences specific to kinases PKC-α, PKA, PAK and CK2, alongside a control possessing a poly-glycine sequence (Fig. 6 and Supplementary Table 4). The original ΔDIAPH1 remains the most strongly phosphorylated sequence. While ΔDIAPH1contains an Ala instead of a basic residue at −3, it possesses a Leu at +2 and a Ser at +4 in accordance with the consensus motif compiled from KISS. Phosphorylation is slightly attenuated with basic substitutions at −2 and −3 as with G1-15-PAK. This is followed by G1-15-PKC-α and G1-15-PKA, which are phosphorylated at about half the intensity to G1-15-ΔDIAPH1. There is very weak phosphorylation of G1-15-CK2 and almost no phosphorylation of G1-15-poly-G. Substitution of residues +2 to +4 with acidic residues as with G1-15-CK2 strongly attenuates phosphorylation. These patterns suggest that generally, YopO prefers basic or hydrophobic residues both up and downstream of the phosphorylation site.

## Discussion

Protein phosphorylation is a universal mechanism for regulating cellular processes in eukaryotes. Pathogenic *Yersinia*, through the injection of T3SS effectors into immune cells, competes for control of host signaling events to prevent clearance and achieve dissemination and infection. Here we describe and establish a greater understanding of the mechanism of action of YopO, a serine/threonine kinase from pathogenic *Yersinia*, and show that it phosphorylates eukaryotic actin cytoskeleton targets, revealing the possible contributions of YopO’s kinase activity in *Yersinia* pathogenicity.

Structural analysis of the kinase domain of YopO has revealed closer similarity to eukaryotic kinases with respect to bacterial kinases. A structure similarity search yielded PAK1 and MST3 kinase domains as the most similar structures to the YopO kinase domain. Coincidentally, PAKs play important roles in actin cytoskeletal organization and bind to small GTP-binding proteins, albeit through different mechanisms. PAKs bind to Cdc42 and Rac1-like GTPases through their N-terminal CRIB motifs, which are structurally distinct to the GDI domain of YopO, while their kinase domains lie at the C-termini. PAKs when activated by Rho GTPases, phosphorylate substrates including myosin light-chain kinase (MLCK), cortactin, LIMK1 and filamin A to promote the formation or stability of the polymerized actin-rich structures found in lamellipodia and filopodia^29^. MST kinases are regulators of the Hippo pathway, and contain a kinase domain followed by a bipartite nuclear localization and export signals. MST kinases, unlike the PAKs, are not the direct targets of small G-proteins. The conformational similarity of the kinase domain of YopO with these eukaryotic kinases supports the hypothesis that bacterial Ser/Thr kinases may have arisen by a process that included horizontal gene transfer from ancestral eukaryotes^30^.

The KISS proteome-wide screen revealed that YopO specifically phosphorylates actin-modulating proteins. Competition assays with G1 and profilin on a subset of these proteins in this and our previous work^16^ demonstrated that phosphorylation is dependent on their recruitment by YopO-actin. This suggests that being able to bind actin either directly or indirectly serves as an important selection criterion. Navarro’s group has shown that YopO binds to and phosphorylates Gαq, the heterotrimeric G protein complex α subunit^31^. We did not detect either Gαq or its phosphorylated sequences in KISS. Structural similarity of the kinase domains of YopO and PAK, their possession of a Rho GTPase targeting domain and that PAKs are also cytoskeleton regulators prompted us to investigate if YopO is mimicking PAKs in cytoskeletal regulation. However, amongst the substrates that were tested, YopO shares only one substrate with PAKs and that is SHOT1. This reveals that despite their structural similarity, YopO and PAKs have different substrate requirements.

As YopO phosphorylates only a subset of direct actin binders, we went on to establish characteristics that identify whether an actin-binding protein will be phosphorylated by YopO. The artificial substrates suggest that YopO has a minimum length requirement from where the actin-binding proteins bind to actin in order to reach into the kinase catalytic cleft. Above which, there appears to be no maximum length restrictions and this may allow YopO to scan across the length of a protein for sites that can be phosphorylated. Compilation of YopO phosphorylation sites across the substrates revealed that YopO strongly prefers the phosphorylation of serine over threonine residues, as found for PKA^32^ and PAKs^33^. The consensus sequence derived by KISS and these artificial substrates appear to agree, with basic or small hydrophobic residues at −3 and small hydrophobic residues at +2 and +3. The artificial substrates with consensus sequences for other kinases suggest that YopO may recognize sites targeted by PAK, PKC and PKA, containing basic residues upstream of the phosphoacceptor. Such substrate preferences are shared by a large number of other kinases, including AMPK, PKB, ZIP kinase, PIM1 and RSK1. These data suggest that YopO may target phosphorylation sites on actin-modulating proteins recognized by a range of kinases. These actin-modulating proteins may directly bind actin, or be recruited through the formation of complexes with actin binders.

During pathogen detection and clearance by phagocytosis in professional phagocytes, particle-receptor recognition induces the recruitment of actin regulators to the site of ingestion where actin polymerization serves as the driving force for engulfment^34^. Here we report that YopO phosphorylates a number of actin-modulating proteins, encompassing diverse functionalities, some of which have critical roles in orchestrating phagocytosis. EVL and VASP, localized at focal adhesions, filopodia and lamellipodia, function as actin filament elongation factors^35^ and are involved in FcγR-mediated phagocytosis, accumulating with F-actin at nascent FcγR-induced phagosomes^36^. DIAPH1 and INF2 are formins, and serve to assemble unbranched actin filaments^37^. Formins are present in actin-rich podosomes and phagocytic cups of macrophages^38^ and DIAPH1, in particular, is involved in CR3-mediated phagocytosis in macrophages^39^. The mechanism of SHOT1 in immune cell function is less well understood. In the brain, it serves as an actin-binding protein involved in the formation of neuronal polarity and axon outgrowth^40^. It is also present in a wide range of tissues, colocalizing with E-cadherin and cortactin at cell-cell contacts^41^. CAP1 is involved in actin turnover, recycling ADF/cofilin and actin monomers for sustained cytoskeletal remodelling^42^.

We have shown that YopO phosphorylates a range of actin-modulating proteins with diverse functionalities and have identified their phosphorylation sites. Although we have not determined the implications of the phosphorylation, some of these phosphorylation sites have been previously reported in the native host system, in the absence of YopO. YopO phosphorylates VASP at Ser319 (numbering for mouse, Ser322 in human) which is also targeted by protein kinase D1^43^ and AMPK^44^. AMPK phosphorylates VASP at four sites, Ser153, Ser239, Thr278 and Ser319 (numbering for mouse, Ser157, Ser235, Thr274 and Ser322 in human, respectively) and these modifications reduce the ability of VASP to bind to F-actin filaments^44^. PKD1 phosphorylates VASP at Ser153 and Ser319 (numbering for mouse, Ser157 and Ser322 in human), and in contrast, increases F-actin polymerization and reduces VASP localization at focal adhesions^43^. YopO phosphorylates SHOT1 at Ser249, which is also targeted by PAK1, and results in enhancing SHOT1’s interaction with cortactin at sites of dynamic actin assembly^26^.

Since the majority of YopO’s phosphorylation sites have been reported in proteomic studies of healthy samples and/or cancer cell lines^45^, we hypothesize that YopO hijacks native phosphorylation pathways that exist under non-infected conditions to exert control over actin-cytoskeletal dynamics. In conclusion, we demonstrate that YopO executes a specific strategy to target actin modulating proteins, across multiple functionalities to wrest control of their native signaling, hampering the actin remodeling required for macrophage phagocytosis.

## Experimental Procedures

### DNA constructs and proteins

*Y. enterocolitica* YopO WT (89–729) and SER mutant (89–729, K205Y E206Y E207Y K440Y K441Y), VASP, EVL, DIAPH1, G1 and profilin were expressed and purified following the protocols described previously^16^. CAP1 was the mouse protein while VASP, EVL, SHOT1 and profilin were from human and these were expressed as full-length proteins. The DIAPH1 construct (mouse, residues 583–1255) was a gift from L. Blanchoin and was expressed in bacteria with an N-terminal glutathione S-transferase (GST) and a C-terminal His6 tags and purified as previously described^16^. CAP1 was a gift from P. Lappalainen. SHOT1 and CAP1 were expressed in bacteria with an N-terminal His8-tag and purified by Ni-NTA affinity chromatography, cleaved with 3C protease and subjected to size-exclusion chromatography. Both CAP1 and SHOT1 degrade rapidly to give rise to multiple bands on SDS-PAGE. Actin was purified from Sf9 cells with GST-tagged gelsolin domains G4–G6, as previously described^16^. PAK4, encompassing the catalytic domain (278–591), a gift from E. Manser, was expressed in bacteria with an N-terminal His8-tag and purified using gradient Ni-NTA affinity chromatography. The artificial substrates G1-ΔDIAPH1 with different linker lengths were generated using overlap extension PCR and mutated into different consensus sequences using site-directed mutagenesis. The artificial substrates were expressed in bacteria with an N-terminal His8-tag and purified using HisTrap FF columns (GE Healthcare) and eluted via cleavage with 3C protease.

### Crystallization and data collection of YopO-actin in complex with Mg^2+^ and ATP

A surface entropy reduction (SER) version of YopO (K205Y, E206Y, E207Y, K440Y, and K441Y) was prepared and complexed with Sf9 actin and crystallized^16^. Harvested crystals were soaked in 0.1 M HEPES, pH 7.5, 30% PEG400, 0.2 M NaCl, 5 mM MgCl_2_, 5 mM ATP, 25% glycerol for 30 min followed by flash freezing in liquid nitrogen. X-ray diffraction data for YopO-actin:MgATP crystals were collected at the Diamond Light Source (DLS) from a single crystal. Data were recorded at a wavelength of 1 Å, using a Pilatus3 6 M detector on beamline I03 at 105 K.

### Structure determination, model building and refinement of YopO-actin: MgATP

Crystallographic data were indexed, integrated and scaled using xia2 (Winter *et al*. 2013; Kabsch 1993). Various crystallographic software packages were employed in the structure determination process that included CCP4 programs^46^, PHENIX^47^, and COOT^48^. Molecular replacement was performed using Phaser^49^ as part of the PHENIX suite of crystallographic programs, using the YopO-actin apo coordinates^16^ as a search model. Restrained positional and B-factor refinement were performed using phenix.refine^50^ with the apo structure of YopO:actin as a reference model. Loops were built with the use of Phenix fit_loops^51^. Manual building was performed using the graphics program COOT. MolProbity, from PHENIX and validation tools in COOT were used to assess the quality of the model during the refinement of the structure. After several rounds of refinement by the program Phaser^49^, ligands were modeled using PHENIX LigandFit and metal-binding sites were assessed with the CheckMyMetal web server^52^. The final structure was optimized with PDB_REDO^53^. 97% of the residues were in the most favored regions of the Ramachandran plot, 3% were in the additionally allowed regions, and there were two outliers. The data collection and refinement data are given in Table S1.

### Modified KISS

Commercially available immortalised mouse cell line RAW264.7 was used and all methods and experimental protocols were carried out in accordance with the IMCB Institutional Biosafety Committee (IBC) guidelines and regulations. Heavy’ and ‘light’ lysates were prepared from Raw264.7 cells grown either in heavy isotopic [13C6]arginine and [13C6]lysine or in normal isotopic [12C6]arginine and [12C6]lysine (Cambridge Isotopes), respectively. His8-tagged YopO immobilized on Ni-NTA beads was used to pull down binding proteins from ‘heavy’ lysates for 3 h at 6 °C, while in parallel His8-tagged YopO KD was used against ‘light’ lysates. After extensive washes, the beads were incubated with kinase buffer containing 20 mM HEPES, pH 7.6, 1.0 mM ATP, 1 mM DTT, 10 mM MgCl_2_, and 2 mM MnCl_2_. The phosphorylation reactions were incubated for 30 min at 30 °C. Bound proteins were eluted by denaturation with 20 mM HEPES, pH 8.0, 8 M Urea.

### Tryptic digestion for modified KISS

1/10 volume of 55 mM DTT was added and incubated for 30 min at room temperature. This was followed by the addition of 120 mM iodoacetamide at a volume equal to the DTT solution and incubated for 30 min at room temperature in the dark. The samples were then diluted using 20 mM HEPES (pH 8) to a final concentration of 6 M urea. LysC (Wako) was added with a LysC to protein ratio of 1:100 and incubated at 37 °C overnight. The samples were further diluted to a final concentration of ~1 M urea using 20 mM HEPES (pH 8.0). Sequencing-grade trypsin (Promega) was added with a trypsin to protein ratio of 1:50 (w/w). After 4 hours of digestion at 37 °C, the peptide solution was cleaned up using a C18 cartridge (3 M™ Empore™, 3 mL).

### IMAC Beads Preparation for Modified KISS

IMAC beads were prepared as described^54^ with minor modifications. Briefly, 200 μl of Ni-NTA agarose conjugates (Qiagen) was rinsed thrice with 800 μl MilliQ water. 800 μl of 100 mM EDTA pH 8.0 was then added with incubation for 30 minutes at RT to strip the nickel. Residual nickel was rinsed 3 times with 800 μl MilliQ water, followed by incubation with 800 μl of a 100 mM iron chloride solution for 30–45 minutes. The resin was washed 3 times with 800 μl MilliQ water, followed by rinsing 4 times with 800 μl 80% acetonitrile/0.1% trifluoroacetic acid.

### Phosphopeptide Enrichment for Modified KISS

The tryptic peptides were then enriched using the iron chloride treated IMAC beads. Tryptic peptides were reconstituted in 600 μl 50% acetonitrile/0.1% trifluoroacetic acid, followed by 1:1 dilution with 100% acetonitrile/0.1% trifluoroacetic acid. The peptides were incubated with 10 μl of IMAC beads for 30 minutes with end-over-end rotation. The beads were then placed into a self-packed C18 StageTip^55^, which was pretreated with 200 μl methanol, washed with 100 μl 50% acetonitrile/0.1% formic acid and equilibrated with 200 μl 1% formic acid. Following loading onto the stage tip, the IMAC beads were washed with 100 μl 80% acetonitrile/0.1% trifluoroacetic acid and 200 μl 1% formic acid. The phosphopeptides were eluted from the IMAC beads onto C18 membranes using 4 × 70 μl 500 mM dibasic sodium phosphate (pH 7.0), followed by washing using 200 μl 1% formic acid. The phosphopeptides were stored on the stage tip until they were ready to be analyzed by LC-MS. The phosphopeptides were eluted from the C18 membranes with 60 μl 50% acetonitrile/0.1% formic acid, dried using a speedvac and reconstituted in 24 μl of 0.1% formic acid.

### LC/MS Analysis for Modified KISS

Reconstituted peptides were analyzed using an EASY-nLC 1000 (Proxeon, Fisher Scientific) attached to a Q-Exactive (Thermo Fisher Scientific). Peptides were enriched using a C18 pre-column and separated on a 50 cm analytical column (EASY-Spray Columns, Thermo Fisher Scientific) at 50 °C using a 265 min gradient ranging from 0 to 40% acetonitrile/0.1% formic acid, followed by a 10 min gradient ranging from 40 to 80% acetonitrile/0.1% formic acid and maintained for 10 min at 80% acetonitrile/0.1% formic acid. Survey full scan MS spectra (m/z 310–2000) were collected with a resolution of r = 70,000, an AGC target of 3e^6^ and a maximum injection time of 10 ms. Twenty of the most intense peptide ions in each survey scan with an intensity threshold of 10,000, underfill ratio of 1% and a charge state ≥2 were sequentially isolated with a window of 2 Th to a target value of 50,000 with a maximum injection time of 50 ms. These were fragmented in the high energy collision cell by dissociation, using a normalized collision energy of 25%. The MS/MS was acquired with a starting mass of m/z 100 and a resolution of 17,500 and dynamic exclusion of duration of 15 s.

### Phosphorylation and tryptic digestion for *in vitro* phosphorylation site determination

DIAPH1, VASP, EVL, SHOT1 and CAP1 were incubated in separate reactions with YopO WT, Sf9 actin and profilin in a ratio of 5:1:1:1 for 30 min at 30 °C in kinase buffer (20 mM HEPES, pH 7.6, 1.0 mM ATP, 1 mM DTT, 10 mM MgCl_2_, 2 mM MnCl_2_) for the phosphorylation reaction to take place. The phosphorylated substrates were run on a NuPAGE 4–12% Bis-Tris Gel (Invitrogen), excised, and then subjected to reduction, alkylation and in-gel digestion via the following protocol. Gel pieces were washed with 50 μl of 50 mM ammonium bicarbonate. The gel pieces were reduced in 10 mM DTT for 30 min at 56 °C. Alkylation was carried out away from light, with 55 mM iodoacetamide for 20 min at room temperature. The gel pieces were washed in 50 μl of 50 mM ammonium bicarbonate and shrank twice for 10 min in 50 μl of 100% acetonitrile. 30 μl of 13 ng/μl sequencing-grade trypsin (Promega) was added to each well for 30 min at 4 °C before addition of 25 mM ammonium bicarbonate to cover the gel pieces for incubation overnight at 37 °C. The supernatant-containing peptides were collected following centrifugation. For further peptide extraction, 20 μl of 5% formic acid was added to each well followed by 20 μl of 100% acetonitrile, twice.

### LC/MS analysis for *in vitro* phosphorylation

Vacuum dried samples were reconstituted in 0.1% formic acid and analyzed using a nano-HPLC attached to an LTQ Orbitrap classic (Thermo Fisher Scientific). Peptides were adsorbed onto a C18 pre-column and separated on an analytical column using a 4 h gradient with 2 to 40% acetonitrile and 0.1% formic acid, followed by a 5 min gradient with 40 to 80% acetonitrile and 0.1% formic acid. Survey full scan MS spectra (m/z 310–1400) were acquired with a resolution of r = 60,000 at m/z 400, an AGC target of 1e^6^, and a maximum injection time of 1000 ms. Ten of the most intense peptide ions in each survey scan with an ion intensity of >2000 counts and a charge state ≥2 were isolated sequentially with a target value of 5000. These were fragmented in the linear ion trap by collision-induced dissociation using a normalized energy of 35%. A dynamic exclusion was applied with a maximum exclusion list of 500 with one repeat count, 45 s repeat duration and exclusion duration of 30 s.

### Mass spectrometry data processing and database searches

Data were processed using MaxQuant (Version 1.3.0.5)^31^ and searched against Uniprot 2016-06 human or mouse databases containing 262 commonly observed contaminants and sequences of the tagged protein. Database searches were performed with tryptic specificity allowing a maximum of two missed cleavages and two labeled amino acids as well as an initial mass tolerance of 6 ppm for precursor ions and 20 ppm or 0.5 Da for fragment ions analyzed in the Q-exactive or LTQ Orbitrap, respectively. Cysteine carbamidomethylation was searched as a fixed modification, and N-acetylation, oxidized methionine, phosphorylated serine/threonine/tyrosine were searched as variable modifications. Maximum false discovery rates were set to 0.01 for both protein and peptide. Proteins were considered identified when supported by at least one unique peptide with a minimum length of seven amino acids. The best scoring scan for each detected phosphorylation site with an Andromeda Score ≥ 40 were manually validated.

### *In vitro* phosphorylation assay analysis by SDS-PAGE

For the competition assay of CAP1 and SHOT1 with profilin or G1, final protein concentrations were YopO (1.1 μM), G-actin (1.1 μM), substrates CAP1/SHOT1 (4.4 μM), G1 (3.3 μM) and profilin (3.3 μM). For assays involving the artificial substrates, final protein concentrations were YopO (1.8 μM), G-actin (1.8 μM), artificial substrates (5.4 μM). In these, the proteins were mixed and adjusted to 20 μl in 2 mM HEPES, pH 7.6, 0.2 mM ATP, 0.5 mM DTT, 0.1 mM CaCl_2_, and 1 mM Na azide and the reaction was initiated by addition of an equal volume of kinase buffer containing 40 mM HEPES, pH 7.6, 2.0 mM ATP, 2 mM DTT, 20 mM MgCl_2_, and 4 mM MnCl_2_. The phosphorylation reactions were incubated for 30 min at 30 °C and were stopped by the addition of SDS-PAGE sample buffer and heating for 5 min at 95 °C. The SDS-PAGE gels were first stained with Pro-Q Diamond Phosphoprotein Gel Stain (Thermo Fisher Scientific) following the manufacturer’s recommendations and imaged with a Biorad PharosFX plus Molecular imager for visualization of phosphorylated species, followed by Coomassie staining for visualization of total protein.

## Additional Information

**Accession codes:** The coordinates and structure factors of the crystal structure presented in this paper have been deposited in the Protein Data Bank with the accession code 5CE3.

**How to cite this article**: Lee, W. L. *et al*. Mechanisms of *Yersinia* YopO kinase substrate specificity. *Sci. Rep.* **7**, 39998; doi: 10.1038/srep39998 (2017).

**Publisher's note:** Springer Nature remains neutral with regard to jurisdictional claims in published maps and institutional affiliations.

## Supplementary Material

Supplementary Information
