# Supplementary material for: Mechanisms of Yersinia YopO kinase substrate specificity
Source: Sci Rep. 2017 Jan 4;7:39998. doi: 10.1038/srep39998 (PMC5209680; doi:10.1038/srep39998)
Supplement: Supplementary Information [file srep39998-s1.pdf]

SUPPLEMENTARY INFORMATION

Mechanisms of *Yersinia* YopO kinase substrate specificity

Wei Lin Lee, Pavithra Singaravelu, Sheena Wee, Bo Xue, Khay Chun Ang, Jayantha Gunaratne, Jonathan M Grimes, Kunchithapadam Swaminathan and Robert C Robinson

**Data collection**	
Space group	$P2_1$
Cell dimensions	
a, b, c (Å)	108.73, 121.75, 118.58
(α, β, γ) (°)	90.0, 94.9, 90.0
Resolution (Å)	20.0-2.93 (2.97-2.93) [a]
R_{merge}	0.125 (0.778)
I/σ(I)	11.9 (2.7)
Completeness (%)	99.9 (99.8)
Redundancy	6.8 (6.6)
**Refinement**	
Resolution (Å)	2.93-14.69 (2.93-2.97)
No. reflections	64,212
Rwork / Rfree	19.6/24.0
No. atoms	
Protein	14992
Ligand/ion	120
Water	9
B-factors overall ($Å^2$)	75.56
Protein ($Å^2$)	75.72
Ligand/ion ($Å^2$)	56.03
Water ($Å^2$)	57.93
R.m.s deviations	
Bond lengths (Å)	0.006
Bond angles (°)	1.272

Supplementary Table 1: Table of statistics associated with X-ray crystallographic analysis of the YopO-actin:MgADP complex. Data collected from a single crystal. Values in parentheses are for the highest-resolution shell. The Rfree is calculated using 5% of the data.

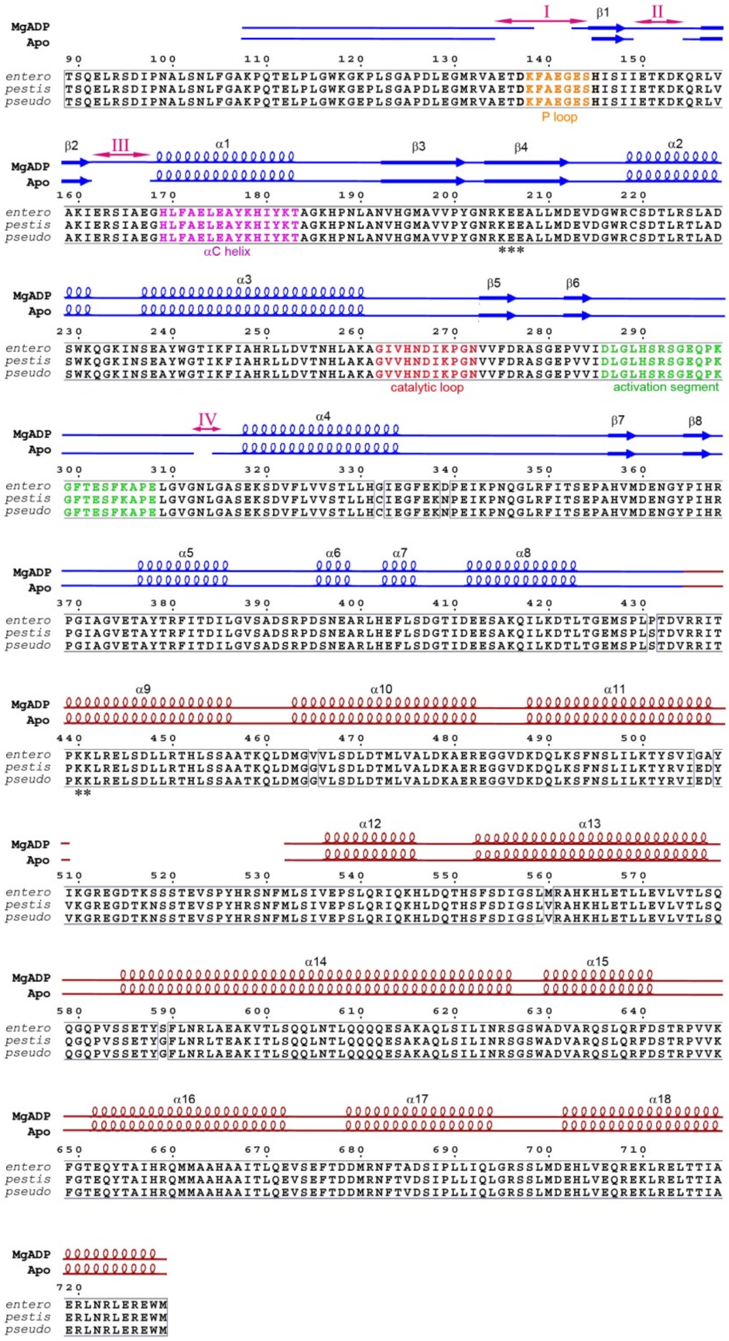

Supplementary Figure 1: The secondary structure alignment of the apo (PDB: 4CI6) and MgADP bound structures of the YopO-actin complex shown alongside the sequence alignment of YopO from different species of *Yersinia.* *Entero*, *pestis* and *pseudo* denote *Y. enterolitica* (Uniprot Q93KQ6-1), *Y. pestis* (Uniprot Q9RI12-1) and *Y. pseudotuberculosis* (Uniprot Q05608-1), respectively. Residues marked with solid blue and yellow squares are involved in the interaction with actin and Rac1, respectively. Residues marked with solid black and grey squares are involved in interactions with Mg^{2+} and ADP, respectively. The regions that were disordered in the apo structure, but are ordered in the MgADP structure, are numbered and labeled with pink arrows.

a

Species	PDB	Chain	Z	rmsd	lali	nres	%id	Description
Homo sapiens	4ZY5	A	23.5	2.6	244	281	16	PAK1 in complex with an inhibitor compound 17
	4QMX	A	23.4	2.8	250	288	17	MST3 in complex with saracatinib
Mycobacteria tuberculosis	1MRU	A	20.4	2.8	228	322	17	PknB in complex with ATP-γ-S
	4Y0X	A	19.4	2.9	237	322	18	PknG in complex with ADP

b

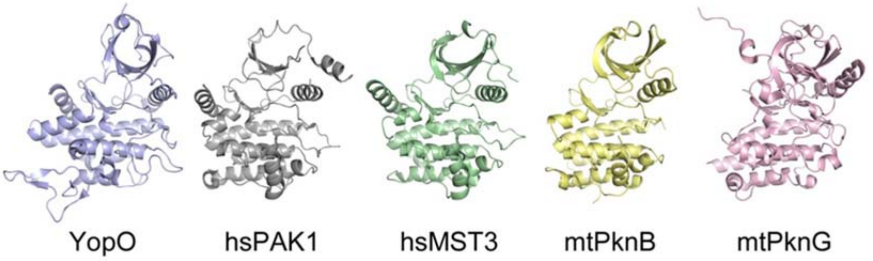

c

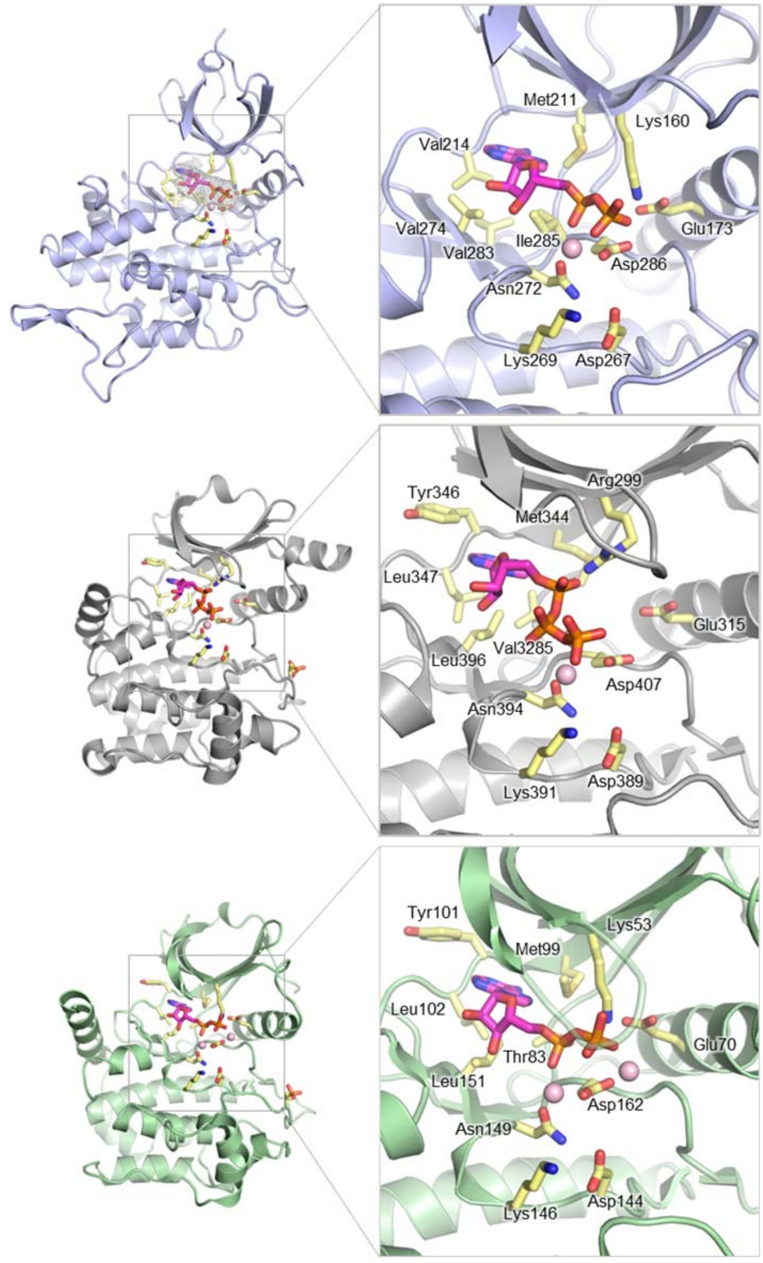

Supplementary Figure 2: Identification of kinases structurally similar to YopO using Dali. (**a**) Table of structurally similar neighbours, showing two of each of the mammalian and bacterial kinases with the highest Z scores. Z score, the statistical significance of the similarity between the protein-of-interest and other neighbourhood proteins; rmsd, root mean square distance, root-mean-square deviation of C-alpha atoms in the least-squares superimposition of the structurally equivalent C-alpha atoms; lali, the number of structurally equivalent residues; nres, the total number of amino acids in the hit protein; %id, percentage of identical amino acids over structurally equivalent residues. (**b**) Visual comparison of structurally similar kinases. hs, *homo sapiens*; mt, *mycobacteria tuberculosis*. (**c**) Structural comparison of the catalytic clefts of YopO, PAK1 (PDB: 3Q53) and MST3 (PDB: 3A7J). The F_o-F_c electron density map at 3.0 Å resolution, contoured at 3.0 σ, before MgADP was built is shown at the active site of the YopO kinase domain. ADP in YopO and MST3 and ATP in PAK1 are represented as sticks. Catalytic residues and residues that line the hydrophobic cleft are also represented as sticks. Mg (YopO and PAK1) and Mn (MST3) ions are shown as spheres (magenta).

a

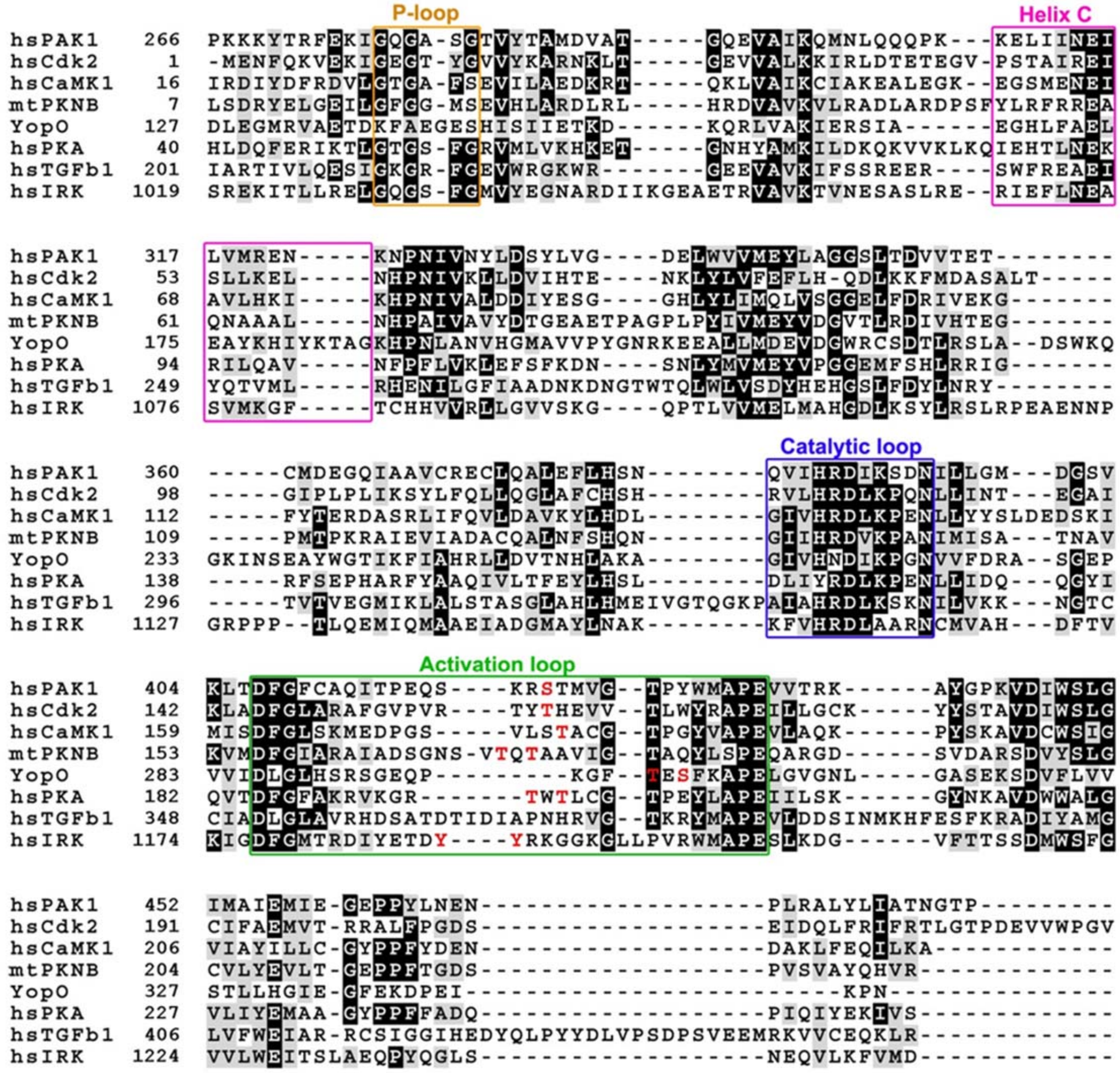

b

Phosphorylation site(s)	Corresponding peptide	No. of phosphates	Charge	m/z	Mass error [ppm]
S90 and S95	GPT(ph)SQELRS(ph)DIPNALSNLFGAK	2	3	825.7154	-1.7258
S95	S(ph)DIPNALSNLFGAK	1	2	763.8662	-1.0731
S102	SDIPNALS(ph)NLFGAK	1	2	763.8662	-1.6329
T301 or S303	GFT(ph)ESFKAPELGVGNLGASEK	1	3	740.0191	-0.36927
	GFTES(ph)FKAPELGVGNLGASEK	1	2	1109.525	0.02749

Supplementary Figure 3: Comparison of YopO against representatives from seven kinase families. (**a**) Structural sequence alignment using PROMALS3D[1]. *Homo sapiens* insulin receptor kinase (hsIRK), *Homo sapiens* transforming growth factor beta receptor 1 (hsTGFb1), *Mycobacteria tuberculosis* protein kinase B (mtPKNB), *Homo sapiens* cyclin-dependent kinase 2 (hsCdk2), *Homo sapiens* p21-activated kinase (hsPAK1), *Homo sapiens* protein kinase A (hsPKA) and *Homo sapiens* calcium/calmodulin-dependent protein kinase (hsCAMK1). Residues in the activation loop that are phosphorylated during activation are shown in red. The autophosphorylation sites on YopO were ambiguously determined and could be either T301 or S303. TGFβ1 is not phosphorylated within the activation loop during activation. (**b**) Autophosphorylation sites identified in YopO by mass spectrometry. An additional two N-terminal residues (Gly and Pro) before T89 of YopO resulted from the 3C protease cleavage site following cleavage. Phosphorylation sites (ph) are indicated.

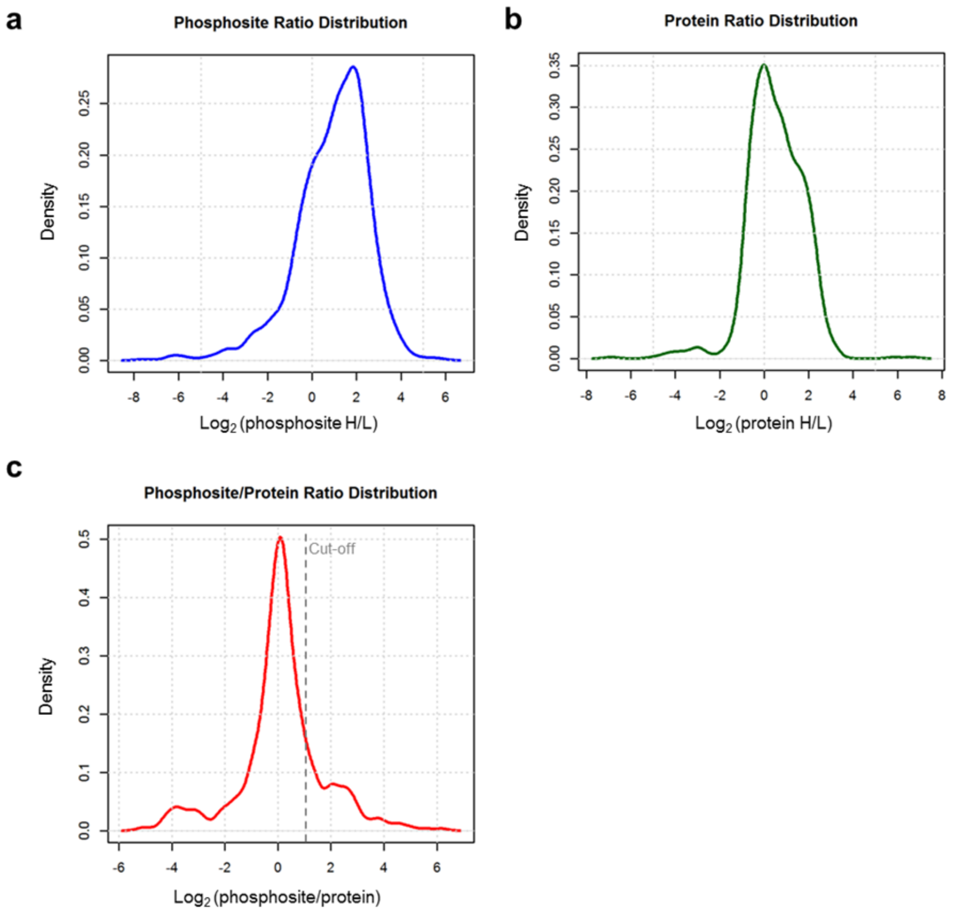

Supplementary Figure 4: Distributions of quantified (a) phosphosites, (b) proteins and (c) the phosphosite/protein ratio. Phosphorylation sites above the peptide/protein ratio of 2 (equivalent to the $\log_2$ value of 1) were considered as putative YopO phosphorylation sites.

	Position (mouse)	Position (human)	Modified sequence	Phosphosite H/L ratio normalized	Protein Ratio H/L normalized	Protein Ratio count	Phospho-site/protein ratio
**Filament elongator**							
**EVL**	S335	S337-p	SNSVEKPVS(ph)SLLSR	31.57	0.94	14	33.45
	S356	S358-p	SPLQS(ph)QPHSR	3.45	0.94	14	3.65
	S360	S362-p	SPLQSQPHS(ph)R	3.70	0.94	14	3.92
**VASP**	S2	S2	(ac)S(ph)ETVICSSR	11.37	0.83	46	13.76
	S285-p	S289-p	ATQVGEKPPKDES(ph)ASEESEAR	1.88	0.83	46	2.27
	S317-p/ S318-p/ S319-p/ S320-p/	S322-p/ S323-p/ S324-p/ S325-p/	S(ph) #S(ph) #S(ph) #S(ph) #VTTSEAHPSTPCSSDDSDLER	1.67	0.83	46	2.01
**DIAPH1**	S1214	S1231	AGCAVTS(ph)LLASELTK	8.13	0.62	20	13.14
**INF2**	S1186	S1158-p	TEADSTSEGPEDEAQRGQS(ph)THLPR	3.54	0.54	9	6.53
**SHOT1**	S249-p	S249-p	RQS(ph)HLLLQSSLPDQQLLK	11.43	1.09	4	10.51
**Actin turnover protein**							
**CAP1**	S254-p	S255-p	S(ph)ALFAQINQGESITHALK	12.14	1.53	57	7.93
	S22/ T24/ S25	S22/ T24/ S25-p	LEAVS(ph)#HT(ph)#S(ph)#DMHCGYGDSPSK	7.76	1.53	57	5.07
**Signalling adaptor**							
**ABI1**	S183-p	S183-p	TNPPTQKPPS(ph)PPVSGR	3.45	1.65	8	2.10
**EPS8**	S57	S57-p	DSVS(ph)SVSDVSQYR	8.57	3.35	11	2.56
**FNBP1L (TOCA1)**	S488-p /S489-p and T496-p /S501-p	S488-p /S489-p and T496-p /S501-p	RHS(ph)#S(ph)#DINHLVT(ph)#QGRES(ph)#PEGSYTDDANQEVR	15.39	5.75	4	2.67
**Scaffolding protein**							
**MTSS1**	S275-p/ S276-p	S271/ S272-p	S(ph)#S(ph)#VCSSLNSVNSSDSR	5.41	0.77	6	7.04
	S279-p	S275-p	SSVCS(ph)SLNSVNSSDSR	10.86	0.77	6	14.12
**PDLIM5 (ENH)**	T110 / S111-p	T110-p / S111-p	EVVKPVPITS(ph)PAVSK	13.38	5.56	4	2.41
**WIPF1**	S330-p	S340-p	NLS(ph)LTSSAPPLPSPGR	1.66	0.60	20	2.77
**Adherens junction protein**							
**PTPN6 (SHP-2)**	S534-p/ Y536-p	S534-p/ Y536-p	GQES(ph)#EY(ph)#GNITYPPAVR	9.95	3.55	5	2.80
**RAVER1**	S524/ S525-p	gap	IPLNPYLNLHSLLPS(ph) #S(ph) #NLAGK	21.16	1.32	9	16.03
	S637-p/ S641-p	gap	S(ph) #GGGS(ph) #GGPLSHFYSGSPTSYFTSGLQAGLK	6.87	1.32	9	5.20
	S696	S531-p	AVGSSPMGSSEGLLGLGPGPNGHS(ph)HLLK	9.36	1.32	9	7.11
	S709/ S711	gap	RS(ph) #FS(ph) #HLLPSPEPSPEGSYVGGQHSQGLGGHYADSYLK	8.05	1.32	9	6.12
**DNA/RNA-binding protein**							
**AHNAK**	S217-p	S216-p	LPSGSGPAS(ph)PTTGSAVDIR	3.24	1.50	7	2.16
**EIF1**	S2-p	S2-p	(ac)S(ph)AIQNLHSFDPFADASK	5.77	0.86	12	6.71
**SF1**	S14	S14-p	(ac)ATGANATPLDFPS(ph)K	9.79	1.29	14	7.57
	S272	S272	S(ph)ITNTTVCTK	9.10	1.29	14	7.03
**UBAP2**	S1018-p	S1004-p	GVS(ph)VSSGTGLPDMTGSVYNK	10.14	2.58	6	3.93
**UBAP2L**	S629-p	S609-p	RYPSSISSS(ph)PQK	6.49	2.54	15	2.55

	S872-p/ S875	S852-p/ S855-p	DGS(ph)#LAS(ph)#NPYSGDLTK	9.64	2.54	15	3.79
ZBP1	T356-p	T371	GEVMESGDSEEPKKEDTGTSSEAT(ph)PPR	7.72	1.96	7	3.95
	S384-p	S405	AMALGDSS(ph)PQTTEPVLR	5.04	1.96	7	2.57

Supplementary Table 2: Phosphorylated peptides identified with KISS. Identified peptides were subjected to cutoffs of mass spectrometry identification score of 44, protein ratio count of 4 and peptide/protein ratio of 2. All cysteines are carbamidomethylated. (ac) denotes acetylation and (ph) denotes phosphorylation. # denotes ambiguous identification in MS due to close proximity of serine/threonine pair.

EVL Position in protein		VASP Position in protein		CAP1 Position in protein		SHOT1 Position in protein		DIAPH1 Position in protein	
**Mouse#**	**Human**	**Mouse#**	**Human**	**Mouse**	**Human#**	**Mouse#**	**Human**	**Mouse**	**Human#**
T19/S20	T19/S20	S2	S2	S22	S22	T14	T14	S844	S861
S64	S64	T4	T4	S48	S49	S101	S101	S942	S959
S243	S245	S8	S8	S90	S91	P124/A125	S124/T125/	T1213	T1230
S249	S251	S9	S9	S254	S255	/T126/T128	T126/T128	S1214	S1231
S251	S253	S34	S34	S265	S266	S191	S191	S1218	S1235
S257	S259	S46	S46	S337	S338	S249	S249	T1242	T1259
S294	S296	P145	S149			S255	S255		
S300	S302	S241	S245			S256	S256		
S316/S317	S318/S319	S254	S258			S351	S351		
S327	S329	S285	S289			S375	S375		
S335	S337	S298	S303			S467	S467		
S356	S358	S300	S305			S473	S473		
S360	S362	S309	S314			S493	S493		
		T310	S315			T496	T496		
		S317/S318	S322/S323			S502	S502		
		/S319/S320	/S324/S325			S504	S504		
						S506	S506		
						S512	S512		
						T551/N552	T551/S552		
						/P553	/S553		
						S561	S561		
						S586	S586		
						S619	S619		

Supplementary Table 3: Phosphorylated peptides identified by mass spectrometry analysis of *in vitro* phosphorylated YopO substrates. Serines or threonines separated by "/" were too close in proximity to differentiate from the mass spectra to identify which site(s) was phosphorylated. Human EVL (Uniprot Q9UI08), VASP (Uniprot P50552) and SHOT1 (Uniprot A0MZ66) and mouse CAP1 (P40124) and DIAPH1 (Uniprot O08808) (residues 583–1255) were used. To facilitate comparison with the phosphorylation sites reported in the literature, corresponding residues in their mouse or human counterparts are shown alongside and denoted with a (#). Shown in grey are corresponding residues in mouse or human which are not conserved.

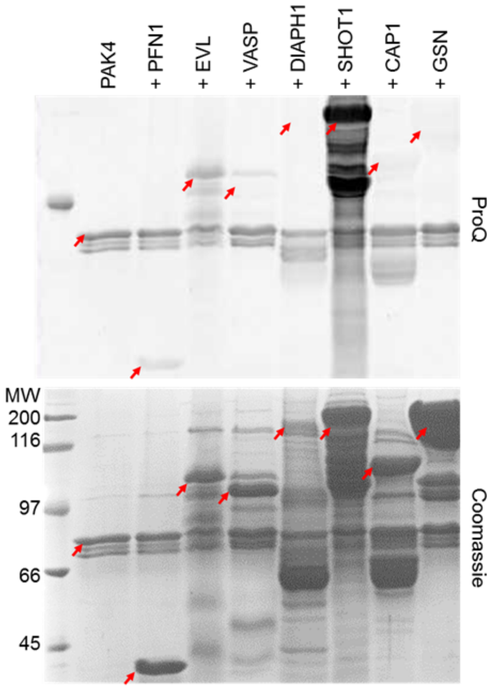

Supplementary Figure 5: *In vitro* phosphorylation of actin-binding proteins by PAK4. The SDS-PAGE was imaged with ProQ Diamond phosphoprotein staining for phosphorylation followed by Coomassie staining for total protein. 3.8 µM of PAK4 was incubated with 15.2 µM of the respective substrates in 50 mM Hepes pH 7.5, 1 mM ATP, 50 mM KCl, 10 mM $MgCl_2$ and 1 mM DTT at 30°C for 30 mins. PFN1, profilin; GSN, gelsolin.

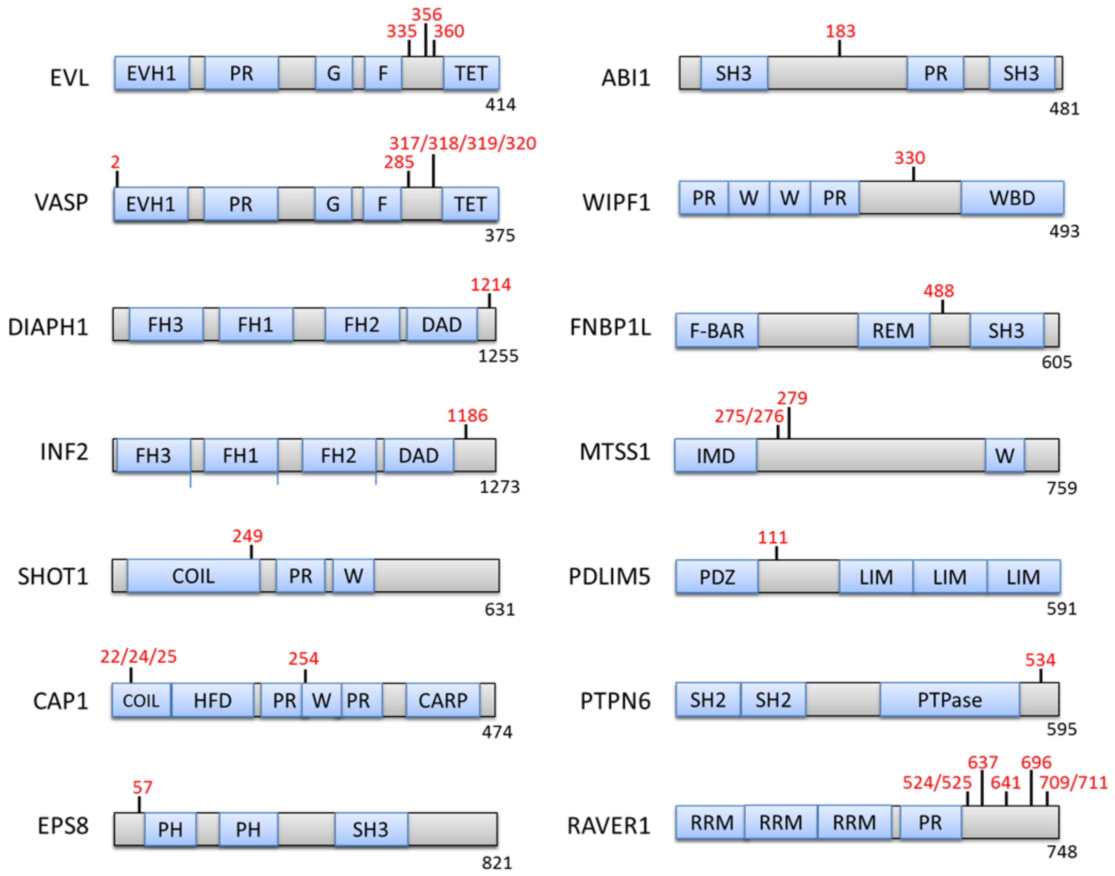

Supplementary Figure 6: Domain organization showing position of validated YopO phosphorylation sites. EVH1, enabled/VASP homology 1 domain; PR, poly-proline; G, G-actin binding domain; F, F-actin binding domain; Tet, tetramerization domain; FH1, 2, 3, formin homology domains 1, 2 and 3; DAD, diaphanous autoregulatory domain; COIL, coiled coil; ; W, Wiskott-Aldrich syndrome homology region 2 (WH2); HFD, helical folded domain; CARP, β-sheet/β-helix (CARP) domain; PH, Pleckstrin homology; SH2 and 3, src homology-2 and 3; WBD, WASP-binding domain; REM, Ras exchanger motif; IMD, IRSp53 and MIM homology domain; PTPase, protein tyrosine phosphatase.

a

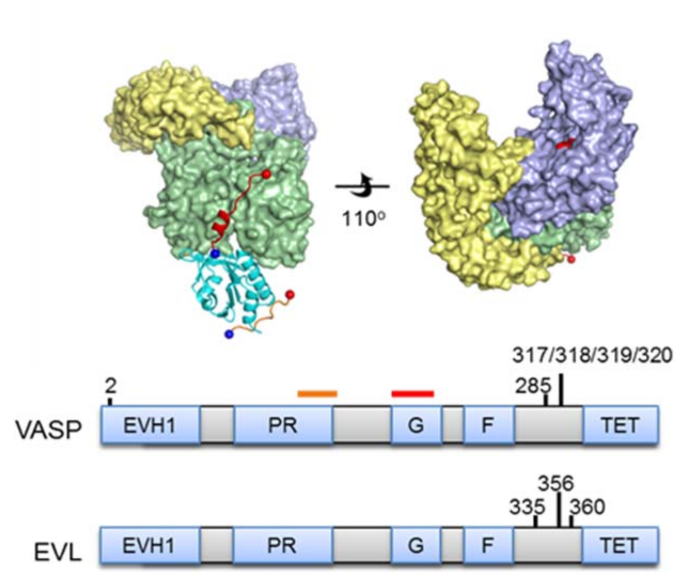

b

```
                       G                                         F
VASP   PGLAAAIAGAKLRKVSKQEEASGGPLAPKA-------ENSRSTGGGLMEEMNAMLARRRK 272
EVL    SGLAAALAGAKLRRVQRPEDASGGSSPSGTSKSDANRASSGGGGGLMEEMNKLLAKRRK 278
        *****:******:*.: *:****     :        .* . ********* :**:***

VASP   ATQVGEKPPKDESAS-------------EESEARLPAQSEPVRRPWEKNSTTLPRMKSSS 319
EVL    AASQTDKPADRKEDESQTEDPSTSPSPGTRATSQPPNSSEAGRKPWERSNSVEKPVSSLL 338
       *:.  :** . :. .              .: :: * .**  *:***:..:.   :.*

                                                         TET
VASP   SVTT-------------------SEAHPSTPCSSDDSDLERVKQELLEEVRKELQKMKE 359
EVL    SRTPSVAKSPEAKSPLQSQPHSRVKPAGSVNDVGLDALDLDRMKQEILEEVVRELHKVKE 398
       * *                    . *   .  . *  **:*:***:**** :**:*:**

VASP   EIIEVFVQELRKRGSP 375
EVL    EIIDAIRQELSGISTT 414
       ***:.: ***   .:
```

Supplementary Figure 7. Model of binding of EVL and VASP to YopO:actin for phosphorylation. Model of the quaternary complex of YopO–actin–profilin–VASP fragment derived from PDB code 2PBD, in which the poly-proline sequence is shown in orange and the G-actin binding domain is in red. The N- and C-termini are shown as blue and red spheres respectively. The model illustrates how the binding of VASP to YopO:actin may allow the region C-terminal of the G-actin binding domain to span into the catalytic cleft for phosphorylation. The similar domain structures of EVL and VASP suggest that they may share a similar mode of binding, though this does not explain the phosphorylation observed on Ser2 of VASP. Mouse orthologs are shown here with the KISS phosphorylation sites shown in bold red. Residues shown in red but not in bold are ambiguously identified and either one of the four serines could be phosphorylated. G, G-actin binding domain; F, F-actin binding domain; Tet, tetramerization domain.

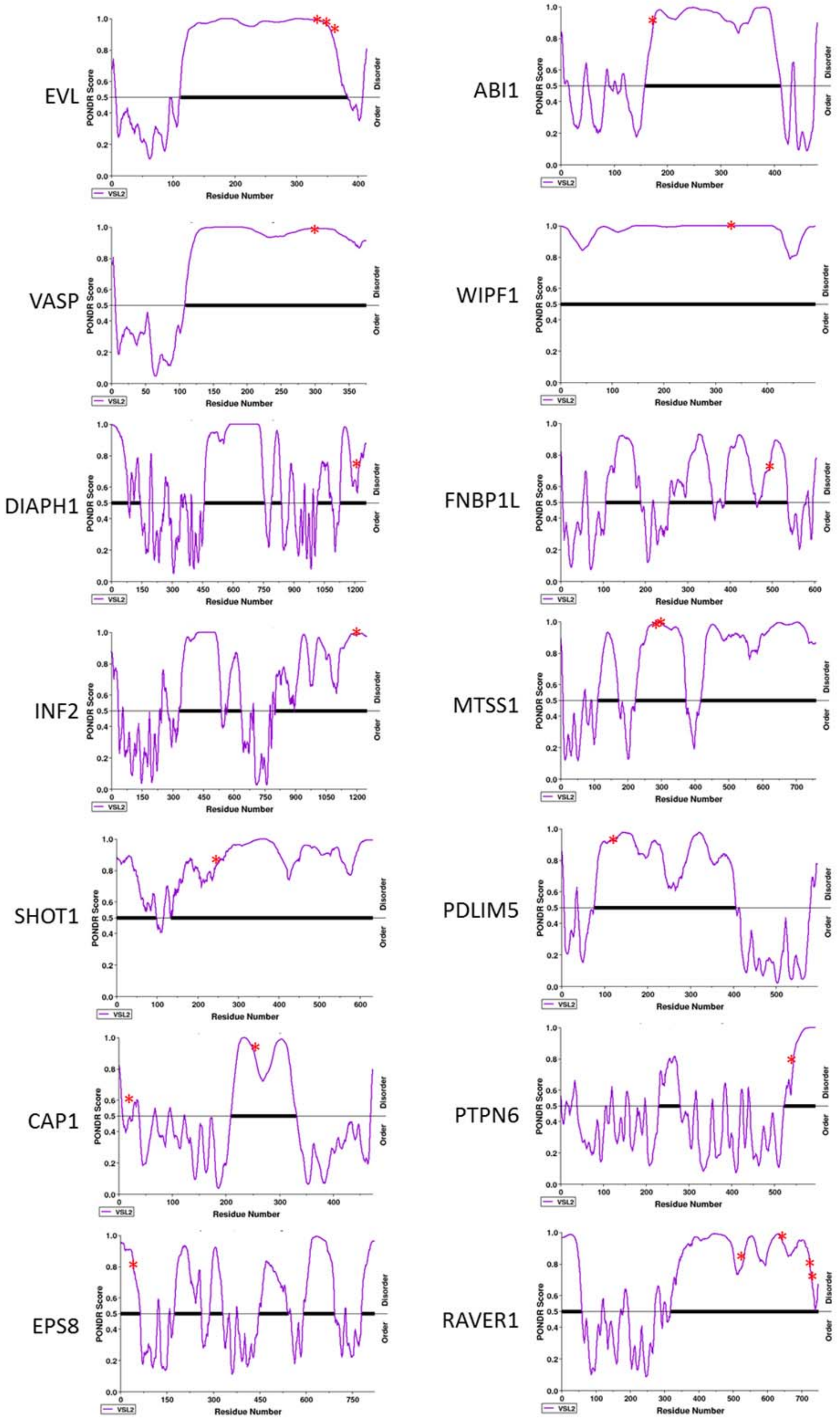

Supplementary Figure 8: Prediction of disordered regions on YopO substrates using PONDR. The prediction was performed on mouse orthologs using the VSL2 predictor algorithm. Residues phosphorylated by YopO are indicated by red asterisks.

	In vitro phosphorylation sites	KISS sites
**EVL**	QQVVINYSIVKGLKY<br>PEDASGGSSPSGTSK<br>GSSPSGTSKSDANRA<br>SPSGTSKSDANRASS<br>KSDANRASGGGGGGL<br>AEKKEDESQMEDPST<br>ESQMEDPSTSPSPGT<br>GRKPWERSNSVEKPV<br>NSVEKPVSSILSRTP<br>EAKSPLQSQPHSRMK<br>PLQSQPHSRMKPAGS	NSVEKPVSSLLSRTP<br>EAKSPLQSQPHSRVK<br>PLQSQPHSRVKPAGS
**VASP**	GTGPQAFSRVQIYHN<br>YHNPTANSFRVVFRV<br>KRQQPGPSEHIERRV<br>VSKQEEASGGPTAPK<br>PKAESGRSGGGGLME<br>EKTPKDESANQEEPE<br>EARVPAQSESVRRPW<br>RVPAQSESVRRPWEK<br>RRPWEKNSTTLPRMK<br>RPWEKNSTTLPRMKS	MSETVICSS<br>EKPPKDESASEESEA
**DIAPH1**	SKTAQNLSIFLGSFR<br>NIKPEIVSVTAACEE<br>RKAGCAVTSLLASEL<br>KAGCAVTSLLASELT<br>AVTSLLASELTKDDA<br>KKSEGVPTILEEAKE	KAGCAVTSLLASELT
**INF2**	N.D.	DEAQRGQSTHLPRTG
**SHOT1**	EKQLQLITSLKEQAI<br>NKTLKRISMLYMAKL<br>QEKTVLNSEVLEQRK<br>QNKLKRQSHLLLQSS<br>QSHLLLQSSIPDQQL<br>SHLLLQSSIPDQQLL<br>RVNQSENSVPPPPPP<br>NPIRSLMSMIRKRSH<br>TSSRSLKSLDPENSE<br>KSLDPENSETELERI<br>VTAEADSSSPTGILA<br>EADSSSPTGILATSE<br>PTGILATSESKSMPV<br>GILATSESKSMPVLG<br>LATSESKSMPVLGSV<br>KSMPVLGSVSSVTKT<br>KLEGCTSSKVTFQPP<br>KVTFQPPSSIGCRKK<br>VVVLDPVSTHEPQTK<br>QPENKEDSIENVRET	QNKLKRQSHLLLQSS
**CAP1**	PYVQAFDSLLANPVA<br>RALLATASQCQQPAG<br>SDDSASRSALFAQIN<br>AQINQGESITHALKH<br>VENQENVSNLVIDDT	SDDSASRSALFAQIN
**EPS8**	N.D.	NYARDSVSSVSDVSQ
**ABI1**	N.D.	PPTQKPPSPPVSGRG
**FNBP1L**	N.D.	IRGDRRHSSDINHLV
**MTSS1**	N.D.	SRKSSVCSSLNSVNS
**PDLIM5**	N.D.	VKPVPITSPAVSKVT
**CTNND1**	N.D.	DPRRRLRSYEDMIGE
**PTPN6**	N.D.	N.A.
**Sequence logo**	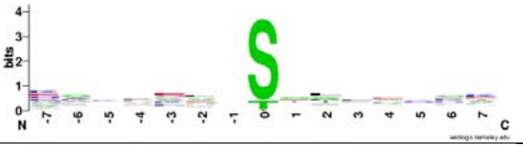	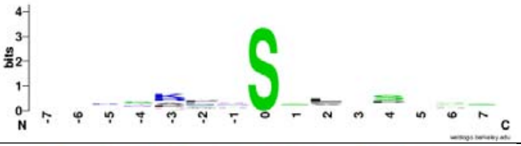
**Frequency plot**	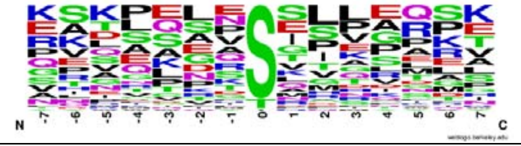	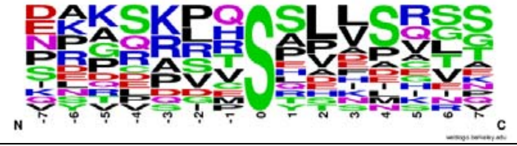

Supplementary Figure 9: Analysis of substrate phosphorylation sequence determinants. Phosphorylation sites including 7 residues up and downstream of the phosphoacceptor as identified by in *in vitro* and KISS phosphorylation were used in the sequence analysis. Ambiguously determined phosphorylation sites were left out from this table and not included in logo generation. *In vitro phosphorylation* was performed using human EVL, VASP, SHOT1 and mouse CAP1 and DIAPH1 and sequences shown are of their respective orthologs. KISS was performed on mouse macrophage-like cell line Raw264.7 and consequently shown sequences were from mouse. The phosphorylated residue is positioned at 0, while positive and negative positions represent amino acids on the COOH- and NH2-terminal side of the phosphorylated residue respectively. These plots are generated by WebLogo[2]. N.D., not determined. N.A., not applicable, due to ambiguous identification.

Constructs	Amino acid sequences	No. of residues between FKHV and phosphoacceptor serine
G1-ΔDIAPH1	GPGRPMVVEHPEFLKAGKEPGLQIWRVEKFDLVPVPTNLYGDFFTGDAYVILKTVQLRNGNLQYDLHYWLGNECSQDESGAAAIFTVQLDDYLNGRAVQHREVQGFESATFLGYFKSGLKYKKGGVASG**FKHV**VPNEVVVQRLKAGCAVTSLLASELT	17
G1-15-ΔDIAPH1	GPGRPMVVEHPEFLKAGKEPGLQIWRVEKFDLVPVPTNLYGDFFTGDAYVILKTVQLRNGNLQYDLHYWLGNECSQDESGAAAIFTVQLDDYLNGRAVQHREVQGFESATFLGYFKSGLKYKKGGVASG**FKHV**VPNEVVVQRLGGAGAGAGGAAGGAGKAGCAVTSLLASELT	32
G1-22-ΔD IAPH1	GPGRPMVVEHPEFLKAGKEPGLQIWRVEKFDLVPVPTNLYGDFFTGDAYVILKTVQLRNGNLQYDLHYWLGNECSQDESGAAAIFTVQLDDYLNGRAVQHREVQGFESATFLGYFKSGLKYKKGGVASG**FKHV**VPNEVVVQRLGGAGAGAGGAGAGAGGAAGGAGKAGCAVTSLLASELT	39
G1-30-ΔDIAPH1	GPGRPMVVEHPEFLKAGKEPGLQIWRVEKFDLVPVPTNLYGDFFTGDAYVILKTVQLRNGNLQYDLHYWLGNECSQDESGAAAIFTVQLDDYLNGRAVQHREVQGFESATFLGYFKSGLKYKKGGVASG**FKHV**VPNEVVVQRLGGAGAGAGGAAGGAGGGAGAGAGGAAGGAGKAGCAVTSLLASELT	47
G1-36-ΔDIAPH1	GPGRPMVVEHPEFLKAGKEPGLQIWRVEKFDLVPVPTNLYGDFFTGDAYVILKTVQLRNGNLQYDLHYWLGNECSQDESGAAAIFTVQLDDYLNGRAVQHREVQGFESATFLGYFKSGLKYKKGGVASG**FKHV**VPNEVVVQRLGGAGAGAGGAAGGAGGGAGAGGGAGAGAGGAAGGAGKAGCAVTSLLASELT	53
G1-15-PKCα	GPGRPMVVEHPEFLKAGKEPGLQIWRVEKFDLVPVPTNLYGDFFTGDAYVILKTVQLRNGNLQYDLHYWLGNECSQDESGAAAIFTVQLDDYLNGRAVQHREVQGFESATFLGYFKSGLKYKKGGVASG**FKHV**VPNEVVVQRLGGAGAGAGGAAGGAGKRRRRKGSFKKSELT	32
G1-15-PKA	GPGRPMVVEHPEFLKAGKEPGLQIWRVEKFDLVPVPTNLYGDFFTGDAYVILKTVQLRNGNLQYDLHYWLGNECSQDESGAAAIFTVQLDDYLNGRAVQHREVQGFESATFLGYFKSGLKYKKGGVASG**FKHV**VPNEVVVQRLGGAGAGAGGAAGGAGKAGKKKRSFIFDELT	32
G1-15-PAK	GPGRPMVVEHPEFLKAGKEPGLQIWRVEKFDLVPVPTNLYGDFFTGDAYVILKTVQLRNGNLQYDLHYWLGNECSQDESGAAAIFTVQLDDYLNGRAVQHREVQGFESATFLGYFKSGLKYKKGGVASG**FKHV**VPNEVVVQRLGGAGAGAGGAAGGAGKAGCKRTSLLASELT	32
G1-15-CK2	GPGRPMVVEHPEFLKAGKEPGLQIWRVEKFDLVPVPTNLYGDFFTGDAYVILKTVQLRNGNLQYDLHYWLGNECSQDESGAAAIFTVQLDDYLNGRAVQHREVQGFESATFLGYFKSGLKYKKGGVASG**FKHV**VPNEVVVQRLGGAGAGAGGAAGGAGKAGCAVTSLDDDELT	32
G1-15-Poly-G	GPGRPMVVEHPEFLKAGKEPGLQIWRVEKFDLVPVPTNLYGDFFTGDAYVILKTVQLRNGNLQYDLHYWLGNECSQDESGAAAIFTVQLDDYLNGRAVQHREVQGFESATFLGYFKSGLKYKKGGVASG**FKHV**VPNEVVVQRLGGAGAGAGGAAGGAGKGGGGGGSGGGGGGT	32

Supplementary Table 4. Protein sequences of the artificial substrates. The residues GPGRP are left after 3C protease cleavage. The FKHV motif, shown in bold, is the gelsolin equivalent of the LKKT motif in WH2 domains[3]. The poly-glycine-alanine linker is shown in red.

References

1 Pei, J. & Grishin, N. V. PROMALS: towards accurate multiple sequence alignments of distantly related proteins. *Bioinformatics* **23**, 802-808 (2007).

2 Crooks, G. E., Hon, G., Chandonia, J.-M. & Brenner, S. E. WebLogo: A Sequence Logo Generator. *Genome Res* **14**, 1188-1190 (2004).

3 Irobi, E. *et al.* Structural basis of actin sequestration by thymosin-β4: implications for WH2 proteins. *Embo J* **23**, 3599 (2004).
